# Manipulating Optical Scattering of Quasi-BIC in Dielectric Metasurface with Off-Center Hole

**DOI:** 10.3390/nano12010054

**Published:** 2021-12-25

**Authors:** Chaobiao Zhou, Tianyao Pu, Jing Huang, Menghui Fan, Lujun Huang

**Affiliations:** 1 College of Mechanical and Electronic Engineering, Guizhou Minzu University, Guiyang 550025, China; putianyao@163.com (T.P.); huangjing@gzmu.edu.cn (J.H.); mhfan@gzmu.edu.cn (M.F.); 2 School of Engineering and Information Technology, University of New South Wales, Canberra 2602, Australia

**Keywords:** dielectric metasurface, optical scattering, bound states in the continuum

## Abstract

Bound states in the continuum (BICs) correspond to a particular leaky mode with an infinitely large quality-factor (Q-factor) located within the continuum spectrum. To date, most of the research work reported focuses on the BIC-enhanced light matter interaction due to its extreme near-field confinement. Little attention has been paid to the scattering properties of the BIC mode. In this work, we numerically study the far-field radiation manipulation of BICs by exploring multipole interference. By simply breaking the symmetry of the silicon metasurface, an ideal BIC is converted to a quasi-BIC with a finite Q-factor, which is manifested by the Fano resonance in the transmission spectrum. We found that both the intensity and directionality of the far-field radiation pattern can not only be tuned by the asymmetric parameters but can also experience huge changes around the resonance. Even for the same structure, two quasi-BICs show a different radiation pattern evolution when the asymmetric structure parameter *d* increases. It can be found that far-field radiation from one BIC evolves from electric-quadrupole-dominant radiation to toroidal-dipole-dominant radiation, whereas the other one shows electric-dipole-like radiation due to the interference of the magnetic dipole and electric quadrupole with the increasing asymmetric parameters. The result may find applications in high-directionality nonlinear optical devices and semiconductor lasers by using a quasi-BIC-based metasurface.

## 1. Introduction

Scattering manipulation lays the foundation of modern photonics, which has outstanding prospects in wavefront manipulation, optical signal processing, energy collection, and sensing. In recent years, one of the main focuses in nanophotonics is to manipulate the optical scattering in order to obtain novel properties unavailable for traditional optical materials and structures [1,2,3,4,5,6,7,8,9,10].

Metasurfaces, a type of two-dimensional artificial dielectric material composed of subwavelength nanostructures, are able to support multiple multipole oscillations at the same time, and the interference of multipoles determines the resonant characteristics and far-field distributions [11,12,13,14,15,16,17]. Unlike the metal metasurface mainly supporting the electric dipole oscillation, there are abundant magnetic responses in all-dielectric metasurfaces, enabling the manipulation of the interference between the electric dipole, magnetic dipole, toroidal dipole, and other higher-order multipoles [18,19,20,21,22,23,24,25,26,27,28,29,30,31,32,33,34,35,36,37,38,39]. The investigation of multipole interference is of great significance to further exploring resonance mechanisms and manipulating radiation characteristics. For example, when perpendicular electric and magnetic dipoles are excited simultaneously and are of equal intensity, unidirectional scattering can be formed by interference between them, thus enhancing the forward scattering and suppressing the backscattering, and vice versa. This is known as the Kerker effect [40,41,42]. Another example of multipole interference is the formation of the anapole mode, which has strong near-field enhancement but suppressed far-field radiation. The physical mechanism behind this is the destructive interference of electric dipoles and toroidal dipoles, which shares a similar far-field radiation with the same amplitude and out of phase [43,44,45,46,47].

Bound states in the continuum (BICs) are special nonradiating states with an infinite lifetime [48,49,50,51,52] that were first introduced in quantum systems in 1929 by von Neumann and Wigner [53]. In recent years, BICs are also widely found in all-dielectric metasurfaces [54,55,56,57,58,59,60]. It is noted that, after introducing the symmetry perturbation in nanostructures, the nonradiative BICs can be transformed into radiative quasi-BICs, accompanied by the occurrence of sharp Fano resonances with high Q-factors [61,62,63,64,65,66,67,68], which have been developed in nonlinear optics [69,70,71,72,73,74], imaging and sensing [75,76,77,78], light emission manipulation and lasing [79,80,81], and so on. Quasi-BICs can exhibit a strong coupling of multipoles and may enrich the manipulation of the far-field scattering pattern [82].

In this work, we investigate the scattering characteristics of quasi-BICs in silicon metasurfaces through further exploring the multipole interference. First, the two quasi-BICs with the Fano profile are excited by moving the position of the air-hole in nanodisks. Due to the multipole interference at different wavelengths, the far-field radiation strength and direction vary dramatically near each Fano resonance. Next, we study the influence of the asymmetric parameter *d* and the air-hole radius *r* on the far-field radiation. The calculated results reveal that the radiation strength and directions at the resonance wavelength can be by tuned by the structure parameters. It is noted that, even for the same resonant mode, the multipole scattering pattern can be completely different at various structural parameters. Our results may shed light on developing the photonic devices with the desired radiation pattern.

## 2. Quasi-BICs with Fano Profile Supported by Silicon Metasurfaces

**Figure 1:**
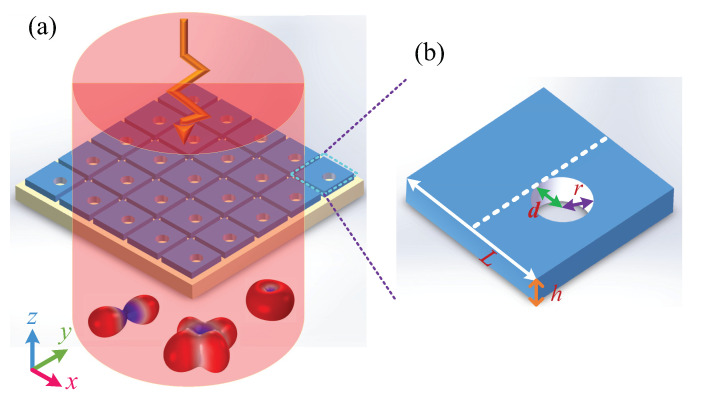
(**a**) Schematic of light radiation manipulated by the resonant metasurface. (**b**) Geometry of unit cell, *L* is the length of the side of a Si square block, *h* is the thickness, *r* is the radius of air-hole, *d* is the distance between the air-hole and the center of the square, which is the asymmetric parameter.

The metasurface is composed of a square array of Si nanodisks with an off-centered round penetrating air-hole lying on a ${\mathrm{SiO}}_{2}$ substrate, as shown in Figure 1. This design can be easily achieved through electron-beam lithography (EBL) and inductively coupled plasma (ICP) etching techniques [80,83,84]. The far-field radiation of incident light can be manipulated by resonant metasurfaces. Figure 1b shows the nanostructure in one unit cell, with a lattice constant *P* of 1300 nm, a side length *L* of 1200 nm, and a thickness *h* of 220 nm. Then, an air-hole is etched from the Si nanodisk to excite resonances. The finite-element method (COMSOL Multiphysics 5.4) is employed to analyze the optical properties of this metasurface. The periodical boundary conditions are set in the *x* and *y* directions and perfectly matched layers are set in the *z* direction. The dielectric constants of Si and ${\mathrm{SiO}}_{2}$ are extracted from the *Palik Handbook* [85]. The air-hole with a radius of 200 nm and a depth of 220 nm is located in the center of each unit cell. A plane wave is normally incident onto the metasurface along the −z direction.

**Figure 2:**
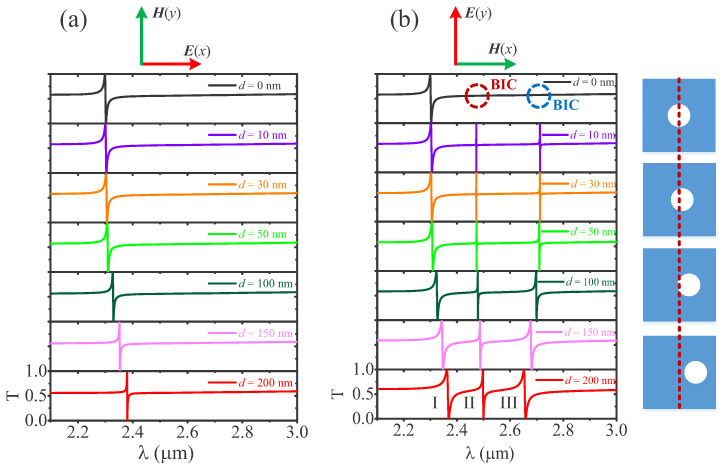
The transmission spectra under (**a**) *x* polarization and (**b**) *y* polarization with different asymmetric parameters *d*.

As illustrated in Figure 2, a polarization-independent, doubly degenerate Fano resonance arises from the constructive and destructive interference of discrete resonance states with broadband continuum states, and the resonance redshifts slightly with the increase in *d*, which is the distance between the air-hole and the nanodisk centers. For the incident light under *y* polarization, however, when the air-hole shifts away from the center position of the Si nanodisk along the *x* direction (or −x direction), the mirror symmetry of the structure along the *y* axis is broken, and two sharper Fano resonances appear to the right of the original resonance peak, indicating that there are two nonradiative symmetry-protected bound states in the continuum (SP-BICs) at d=0. The SP-BIC is produced because the spatial symmetry of the mode does not match that of the external radiation wave, so the mode cannot be radiated. The radiation channel with free space can be constructed by introducing symmetry-breaking into the structure, so that the BIC mode can be converted to a radiable quasi-BIC mode with a high Q-factor. Herein, the off-centered round penetrating air-hole is embedded into Si nanodisks to break the in-plane inversion symmetry of a structure, which can transform the SP-BIC into quasi-BICs with Fano profiles [62,63]. When the offset distance of the air-hole increases, as shown in Figure 2b, which means that the structure becomes more asymmetric, the resonance will have a lower Q-factor due to the increased energy leakage.

**Figure 3:**
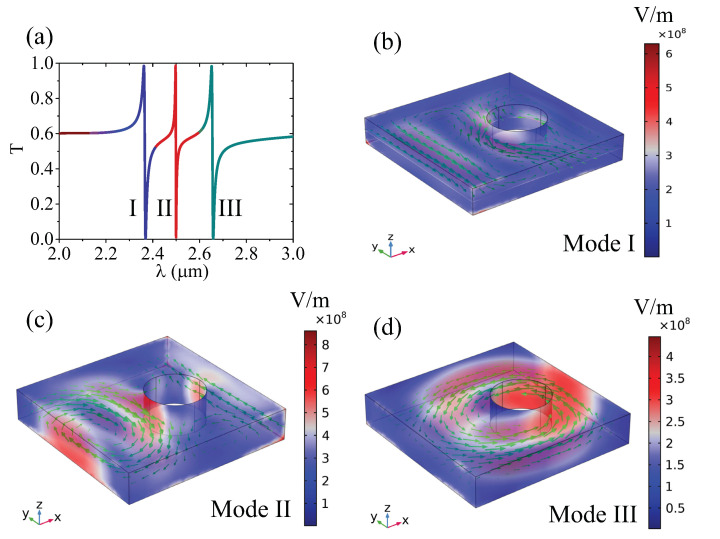
(**a**) Simulated transmission spectra; (**b**–**d**) electric field distribution at the resonant wavelength of modes I, II, and III. Green arrows in x−y plane represent the field direction, and the color scale corresponds to the field intensity.

Next, we fix d=200 nm and r=200 nm to further study the resonance characteristics and far-field radiation. The simulated transmission spectrum is shown in Figure 3a; as discussed above, three resonance modes are observed in the wavelength range of interest, marked as symbols I, II, III. Mode I is a doubly degenerate mode, and modes II, III are the quasi SP-BICs. Figure 3b–d give the corresponding electric field distribution. As illustrated in Figure 3b, the field of mode I is trapped within the structure, and there is no obvious field enhancement at the edge. The field of mode II is enhanced in the off-air-hole region with a circle-distributed field vector, which indicates the bound property of the field. For mode III, the field intensity gets much stronger near the air-hole, and the electric field vector forms an obvious vortex in the x−y plane, indicating that the mode is produced by a strong magnetic dipole oscillation along the *z* direction. It is noted that the resonance spectrum of mode II is relatively sharper with a larger Q-factor, so it supports a stronger field enhancement at the resonance wavelength.

**Figure 4:**
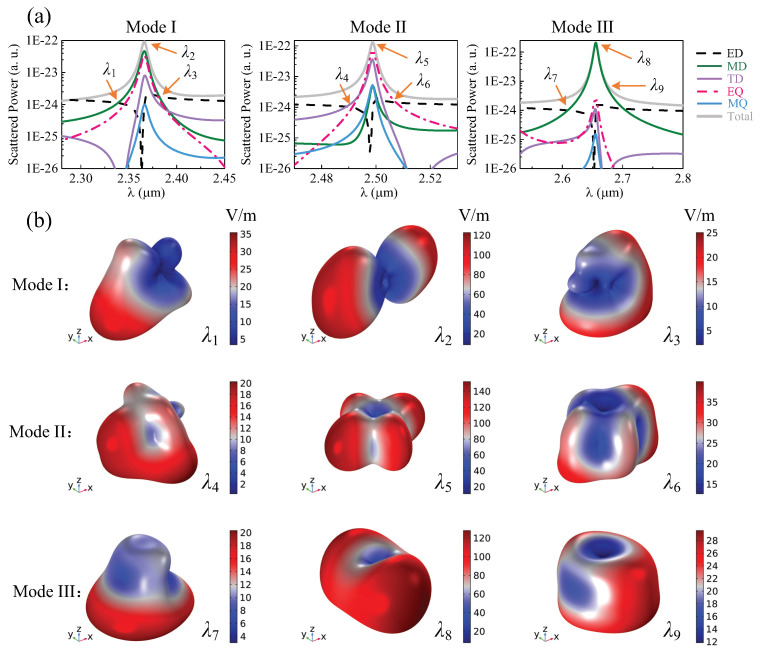
(**a**) The calculated scattered power of different multipoles for modes I, II, and III. (**b**) Radiation patterns of the unit cell at different wavelengths. Here, d=200 nm and r=200 nm.

Multipole decomposition enables an accurate calculation of the multipolar moment and its far-field scattering contribution, which is an effective way to explore the coupling between multipoles. In the following, we perform the multipole far-field scattering of these three resonance modes. First, the electric field in the nanostructure is calculated, which has three dimensions (*x*, *y*, *z*). Then the current ($\vec{j}$) can be obtained through $\vec{j}=-i\omega \epsilon_{0}\left(n^{2}-1\right)\vec{E}$. Finally, the multipole moments and their contributions are calculated by the correlation functions between the multipole moment and displacement current [46,84]. From Figure 4a, we can see that the far-field scattering energy of mode I is mainly contributed by the coupling of the magnetic dipole and electric quadrupole, and that the toroidal dipole also plays a role. The radiation of other multipoles is relatively small. It is noted that mode I is a degenerate state, as illustrated in Appendix A.1. When d=0, the far-field radiation of this mode is dominated by the toroidal dipole. As *d* increases, the moments of other multipoles are manipulated, causing interference between different multipoles. Therefore, the near-field distribution of mode I is perturbed. Compared with d=0, the radiation intensity in the *y*-direction gets weaker at d=200 nm, as shown at $\lambda_{2}$ in Figure 4b. Near the resonance wavelength $\lambda_{2}$, the electric and magnetic dipoles at $\lambda_{1}$ significantly couple to each other (Figure 3a), resulting in distinct unidirectional scattering, as shown in Figure 4b. At wavelength $\lambda_{3}$, the coupling of electric, magnetic, and toroidal dipoles and the electric quadrupole lead to a special far-field radiation pattern, as shown in Figure 4b. For mode II, the far-field scattering at the resonance wavelength $\lambda_{5}$ is mainly contributed by the electric quadrupole, so the radiation pattern displays an obvious quadrupole distribution. The toroidal dipole along the *y*-direction also plays a rule, so the radiation of the quadrupole along the *x*-direction is slightly stronger. At wavelength $\lambda_{4}$, the apparent electric and toroidal dipoles and the magnetic quadrupole coupling results in a scattering enhancement in the *x*-direction. At wavelength $\lambda_{6}$, the electric quadrupole still has a strong scattering, so the far-field radiation shows the property of the quadrupole as well. The electric dipole along the *y*-direction also has a large contribution, so the far-field radiation pattern in the *x*-direction is relatively strong. Due to the fact that $\lambda_{4}$ and $\lambda_{6}$ are in the off-resonance region, the radiation significantly reduces compared to that at $\lambda_{5}$. For mode III, magnetic dipole radiation contributes dominantly at the resonance wavelength $\lambda_{8}$, so an obvious magnetic dipole radiation can be seen in Figure 4b, whose pattern is modified by the coupling of other multipoles. At wavelength $\lambda_{7}$, directional radiation is produced by the interaction between the electric and magnetic dipoles, whereas, at wavelength $\lambda_{9}$, obvious magnetic dipole radiation can be observed. It is noted that the presented radiation pattern is the result of the unit cell of the metasurface, rather than the entire metasurface, while it is still an outcome of the array effect of the metasurface. It is also obviously different from a single nanoparticle, which may support low-Q Mie resonance (as shown in Appendix A.3). The radiation pattern can reveal the interference between multipoles of the resonance mode effectively, which corresponds better with near-field distribution and multipole decomposition.

## 3. Optical Radiation for Different Nanostructure Parameters

### 3.1. Optical Radiation under Different Asymmetric Parameters d

**Figure 5:**
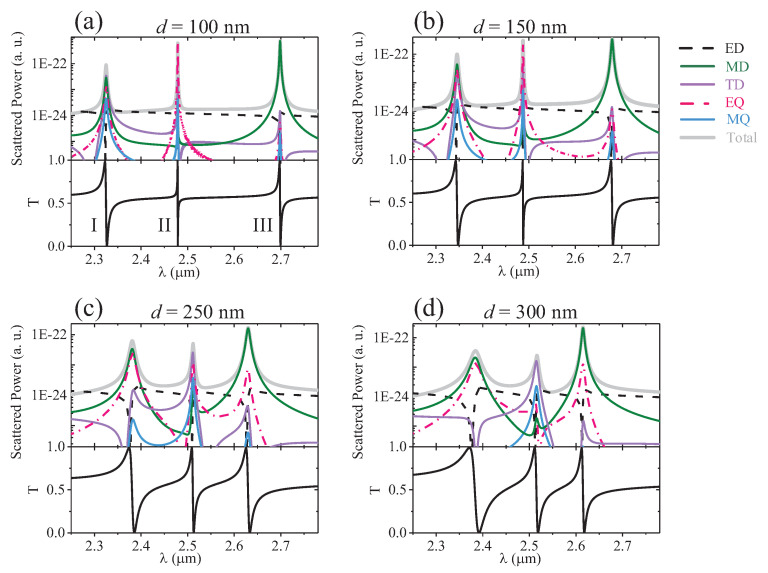
Total scattered power and contributions of different multipoles under different air-hole radii *r*.

**Figure 6:**
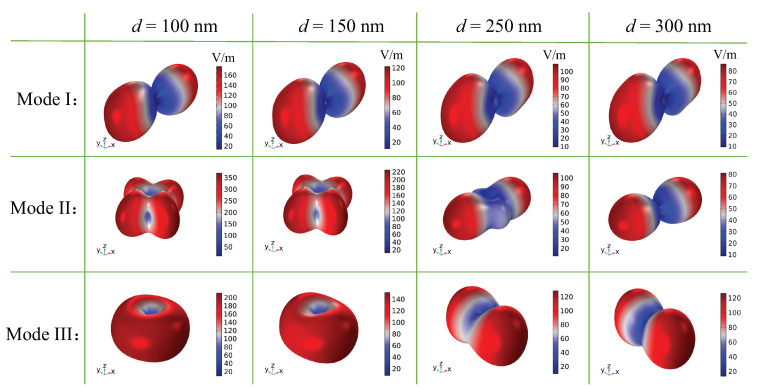
Radiation patterns of the unit cell at different asymmetric parameters *d*.

The far-field radiation of multiple resonant modes has been discussed in the above section. Herein, we investigate the influence of asymmetric parameters *d* on the optical radiation. As shown in Figure 5, for mode I, the resonant scattering at d=100 nm is contributed by the magnetic dipole, magnetic quadrupole, electric quadrupole, and toroidal dipole, with comparable intensity. As *d* increases, the radiation of the magnetic quadrupole and toroidal dipole drops gradually; thus, the total scattering is dominated by the magnetic dipole and electric quadrupole, whereas the corresponding far-field radiation remains approximately unchanged, which is determined by the coupling of the magnetic dipole and electric quadrupole, as shown in Figure 6. For mode II, with the increase of *d*, the mode leakage gets larger and, thus, the resonance gradually becomes broader. The radiation of the electric quadrupole is greatly suppressed. At d=100 nm, the far-field radiation shows an obvious quadrupole pattern, which is mainly contributed by the electric quadrupole. When d=300 nm, the toroidal dipole contributes most to the scattering, which also manifests itself in the radiation pattern. Therefore, by changing the structure parameters, the dipole producing the resonance changes, leading to a different pattern of the far-field radiation, even though the transmission spectrum does not change much. At d=100 nm, the far-field radiation of mode III is mainly contributed by the magnetic dipole, whereas other multipoles are greatly suppressed. The far-field radiation also presents the dipole property. With the increase in *d*, the radiation intensity of the electric quadrupole gets larger. When it interferes strongly with the magnetic dipole, the total radiation along the *x*-direction is inhibited, as shown in Figure 6.

### 3.2. Optical Radiation under Different Radii r of Air-Hole

**Figure 7:**
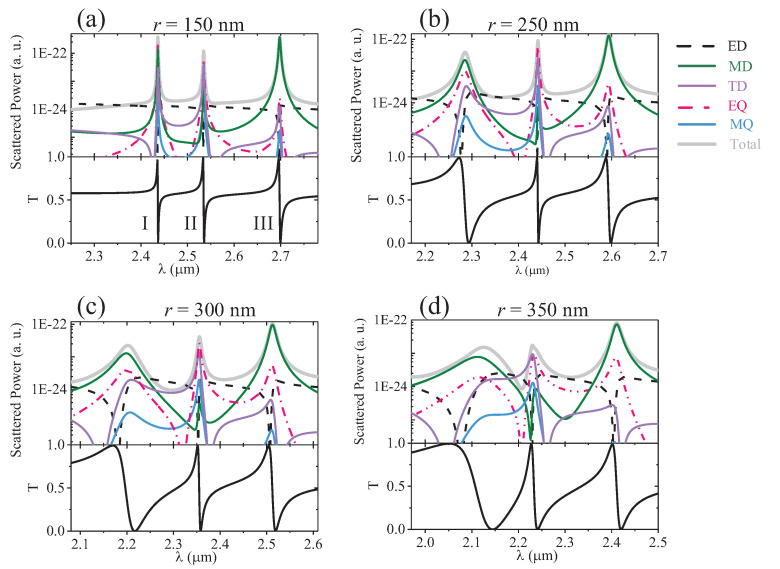
Total scattered power and contributions of different multipoles under different air-hole radii *r*.

**Figure 8:**
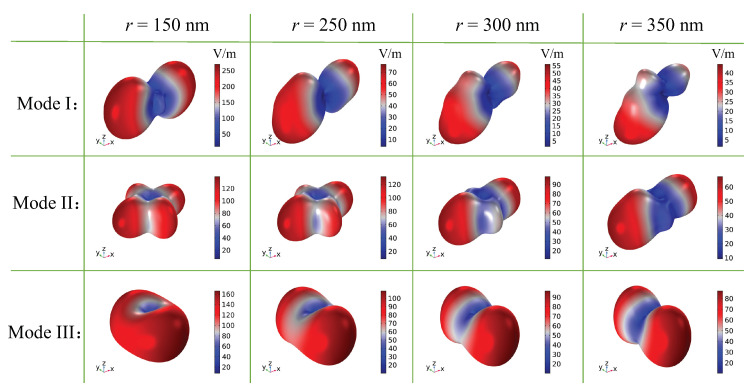
Radiation patterns of the unit cell under different air-hole radii *r*.

Next, we study the dependence of the air-hole radius *r* on the light radiation, as shown in Figure 7. As *r* increases, the resonance spectra of these modes are broadened and their Q-factors decrease. In particular, for mode I, the larger air-hole introduces a larger mode leakage and results in a broader resonance. At the same time, the multipole coupling is modified as well. For mode I, when r=150 nm, the largest contribution to the scattered energy is provided by the electric quadrupole. Due to the influence of the magnetic dipole and toroidal dipole, the radiation strength of the metasurface along the *x*-direction is the strongest, as shown in Figure 8. As *r* increases, the resonance gradually becomes weak, and the outgoing radiation also weakens. At r=350 nm, the scattering intensity of the magnetic dipole reaches the maximum. When it strongly couples with the electric dipole and electric quadrupole, mode I forms directional radiation in the (−x, −z) quadrant. For mode II, when r=150 nm, the far-field scattering of the electric quadrupole is the largest, as shown in Figure 8. With the increase in *r*, the scattering strength of the electric quadrupole and toroidal dipole becomes comparable. The interference between them cancels each other out in the far-field along the *y*-direction, and enhances the radiation along the *x*-direction, as shown in Figure 8. For mode III, with the increase in *r*, the far-field scattering of the magnetic dipole is suppressed, whereas the scattering of the electric quadrupole is gradually improved. Thus, their far-field radiation interferes destructively along the *x*-direction, resulting in the enhancement of the scattering along the *y*-direction.

In general, the symmetry of the structure is disturbed by breaking the geometric structure of the metasurface to excite the SP-BIC, such as split rings [86,87], asymmetric nanorods [78,88], notched cubes [69], and so on. Due to the limitation of fabrication, it is difficult to control its asymmetric parameter in the experiment, so it is difficult to obtain an ultra-high Q-factor by this method. For our work, the symmetry-breaking of the nanostructure is introduced by moving the air-hole position in the nanodisks. In the fabrication process, the specific position parameters of the nanodisks are fixed, and then their lithographic patterns are drawn by L-Edit tool. Finally, the sample is fabricated by EBL and ICP techniques. The fabrication process only changes the uniformity and roughness of the nano-device; the position of the air-hole does not move. Therefore, the asymmetry parameter is accurately controlled in the experiment, which provides a way for the achievement of an ultra-high Q-factor in the SP-BIC metasurfaces.

In addition, our work performs a deep study on the far-field radiation modulation of resonant quasi-BIC modes. In other words, by regulating the interference of multiple dipoles, we can manipulate the far-field radiation pattern and direction of light waves. Therefore, our design provides a route for high-directionality dielectric metasurface lasers and nonlinear optical devices.

## 4. Conclusions

In summary, we study the optical radiation characteristics of multiple quasi-BICs with a Fano profile supported by Si metasurfaces. First, we explore the excitation mechanism of resonant modes and their features. Among them, mode I is a doubly degenerate state, and modes II and III are quasi-BICs excited by the symmetry-breaking of structures. We find that the far-field scattering of these three resonant modes is contributed by the coupling of different multipoles, and shows different properties at the resonant wavelength and nearby wavelengths. Next, we study the influence of the asymmetric parameter *d* and the air-hole radius *r* on the far-field radiation. We find that the radiation intensity of different multipoles is modified dramatically when changing the structure parameters. Thus, even for the same resonant mode, the far-field radiation pattern can vary a lot. For example, with the increase in *d*, the radiation pattern of quasi-BIC mode II changes from electric-quadrupole-dominated to toroidal-dipole-dominated. For quasi-BIC mode III, magnetic-dipole-dominated radiation changes to the directional radiation only along the *y*-direction as *d* increases because of the coupling of the electric quadrupole and magnetic dipole. Our results enrich the tunability of the far-field radiation patterns of a meta-device and may show enormous potential in high-directionality nonlinear optical devices, LEDs, semiconductor lasers, and so on.

## Figures and Tables

**Figure A1:**
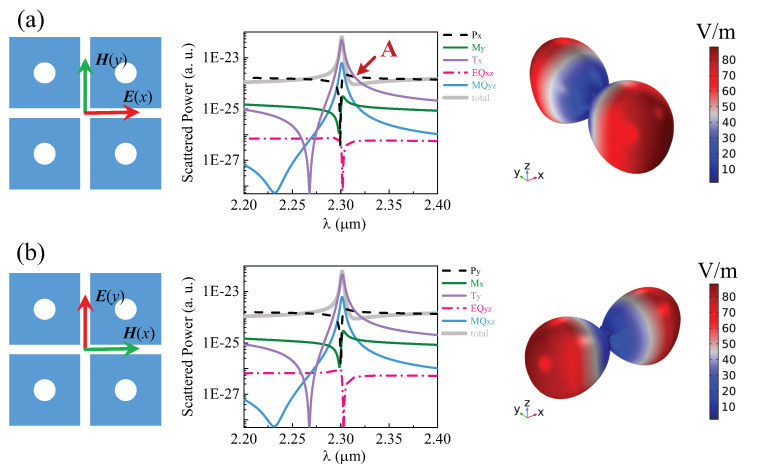
The scattered power of different multipoles and far-field radiation pattern at the resonant wavelength under (**a**) the *x* polarization and (**b**) *y* polarization, respectively.

**Figure A2:**
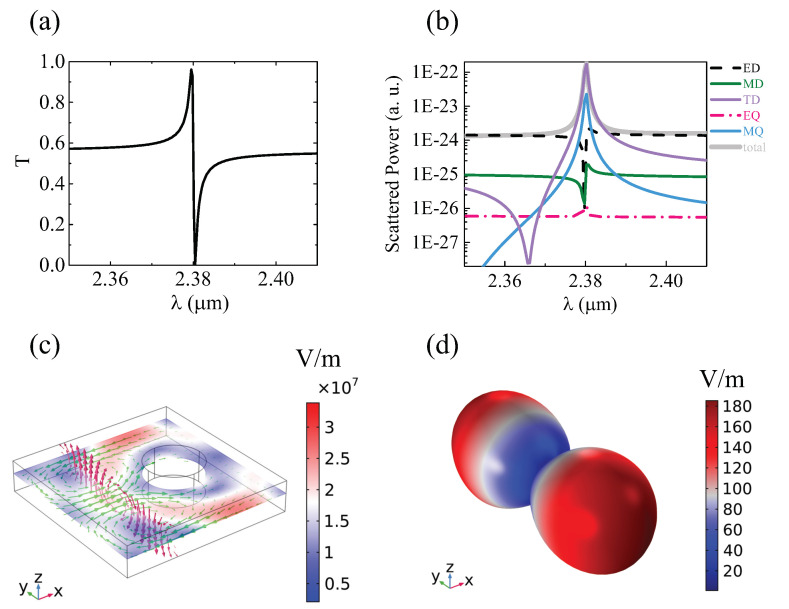
The transmission spectrum, scattered power of different multipole components, electric field distribution, and far-field radiation pattern at the resonant wavelength under the *x*-polarized incidence.

**Figure A3:**
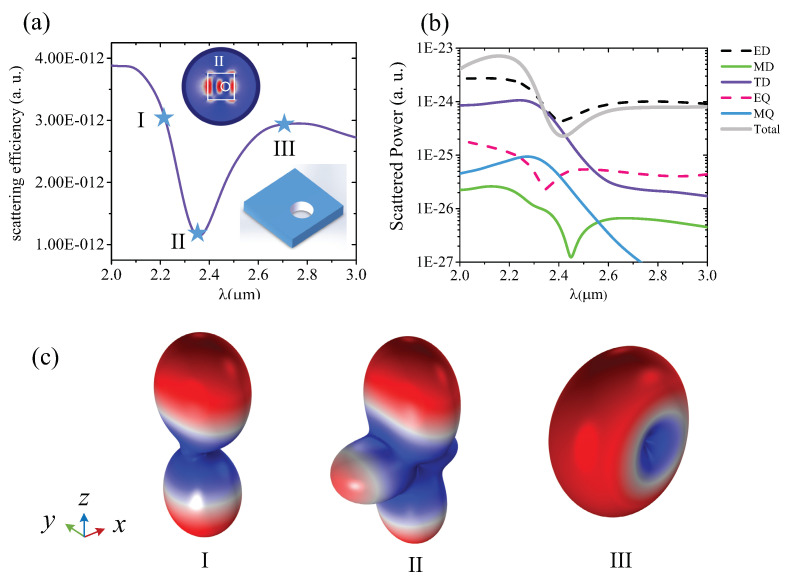
(**a**) Scattering efficiency of isolated nanodisk. The inset shows the electric field distribution of $\lambda_{II}=2.35$ μm, where the corresponding parameter is L=1200 nm, h=220 nm, d=200 nm. (**b**) Total scattered power and contributions of different multipoles. (**c**) Radiation patterns of different wavelengths.

## Data Availability

The data presented in this study are available on request from the corresponding author.

## Appendix A

### Appendix A.1. Optical Radiation at d = 0

When d=0, the metasurface can excite a doubly degenerate mode. Herein, we explore the light radiation characteristics of the metasurface at d=0. The scattered power of multipole components under the *x*-polarized incidence is shown in Figure A1, where the toroidal dipole along the *x*-direction is the dominant contributor. It is noted that there is an obvious contribution from the magnetic quadrupole in the *x*–*z* plane accompanying the dominant toroidal dipole response, because both of them are formed by a pair of counter-oriented magnetic dipoles. Interestingly, at the position A, where the scattering intensities of the electric dipole and toroidal dipole are equal to each other, the total scattering intensity reaches the minimum, which reveals that a weak anapole mode is excited, while the phase difference between these two dipoles fails to reach 2π, so the requirement of an anapole ($\vec{P}=ik\vec{T}$) is not strictly satisfied. As a result of toroidal dipole oscillation along the *x*-direction, the radiation along the *y*-direction is enhanced. Under the *y*-polarized incident light, the far-field scattering intensity and radiation pattern are almost the same as those under the *x*-polarization. The only difference lies in the main direction of radiation due to the different polarization of the incident light. This also shows that the mode is polarization-independent. Based on this, we can manipulate the radiation direction of the metadevice in the *x*–*y* plane by changing the polarization of the incident light.

### Appendix A.2. Optical Radiation under x-Polarized Incidence

In Figure 2b, we observed that mode I is doubly degenerate under the *y*-polarized light. At d=0, the toroidal dipole contributes most to the far-field radiation, whereas, with the increase in *d*, the toroidal dipole radiation is suppressed, and thus the far-field radiation of the resonance is mainly contributed by other multipoles. Since mode I is a degenerate mode, it can also be excited by the *x*-polarized wave. When d=200 nm, r=200 nm; the resonance spectrum is shown in Figure A2a, where the far-field scattering is still dominated by the toroidal dipole, followed by the magnetic quadrupole, as illustrated in Figure A2b at r=0. These results are different from those under the *y*-polarized light. Figure A2c shows the electric field distribution at z=110 nm, where green arrows represent the electric field direction and red arrows represent the magnetic field direction. There are two opposite circular electric field loops in the *x*–*y* plane, which excites a circular magnetic field in the *y*–*z* plane, and finally produces the toroidal dipole along the *x*-direction. Obvious magnetic quadrupole contributions can also be observed accompanying the dominant toroidal dipole response, because both of them are induced by a pair of counter-oriented magnetic dipoles. Figure A2d illustrates the far-field radiation at the resonant wavelength with a great enhancement in the *y*-direction. In a word, the multipole radiation of mode I under the *y*-polarized excitation shows a strong dependence on structural parameters, whereas this dependence is much weaker under the *x*-polarized incidence, which means that mode I is more robust under the *x*-polarized excitation.

### Appendix A.3. Optical Radiation under x Polarized Incidence

It is noted that the resonant modes supported by a single nanoparticle have a lower Q-factor compared to the high-Q modes of the metasurface we study. We perform the calculation of scattering efficiency on a single nanoparticle. From Figure A3a, it can be found that, within the wavelength of the research interest, there is only one low-Q resonance with a weak electric field. This is in stark contrast to that of the three high-Q resonances in the array structure. The emergence of three high-Q resonances can be attributed to the strong lattice coupling. To clarify the physical origin of such a resonance, we perform a multipole analysis on it, and relevant results are presented in Figure A3b. Figure A3c shows the far-field radiation patterns at wavelengths $\lambda_{I}=2.2$μm, $\lambda_{II}=2.35$μm, and $\lambda_{III}=2.7$μm. For $\lambda_{I}$, there is a strong scattering toward the *z* axis, which is the result of the coupling of the toroidal and electric dipoles. For $\lambda_{II}$, the scattered powers of the electric and toroidal dipoles are almost equivalent, while the corresponding phase difference at this wavelength does not satisfy the strict condition of the anapole mode, and the far-field does not have destructive interference completely. With the coupling of other dipoles, the final far-field radiation pattern is shown in Figure A3c. For $\lambda_{III}$; the electric dipole along the *y* axis plays a predominant role and the far-field radiation pattern mainly expands in the *x*–*z* plane.

## References

[1] Jin J., Yin X., Ni L., Soljačić M., Zhen B., Peng C. (2019). Topologically enabled ultrahigh-Q guided resonances robust to out-of-plane scattering. Nature.

[2] Feng T.H., Xu Y., Zhang W., Miroshnichenko A.E. (2017). Ideal Magnetic Dipole Scattering. Phys. Rev. Lett..

[3] Lepeshov S., Krasnok A., Alù A. (2019). Nonscattering-to-Superscattering Switch with Phase-Change Materials. ACS Photonics.

[4] Shcherbinin V.I., Fesenko V.I., Tkachova T.I., Tuz V.R. (2020). Superscattering from Subwavelength Corrugated Cylinders. Phys. Rev. Appl..

[5] Miroshnichenko A.E., Tribelsky M.I. (2018). Ultimate Absorption in Light Scattering by a Finite Obstacle. Phys. Rev. Lett..

[6] Shamkhi H.K., Baryshnikova K.V., Sayanskiy A., Kapitanova P., Terekhov P.D., Belov P., Karabchevsky A., Evlyukhin A.B., Kivshar Y., Shalin A.S. (2019). Transverse Scattering and Generalized Kerker Effects in All-Dielectric Mie-Resonant Metaoptics. Phys. Rev. Lett..

[7] Liu W., Miroshnichenko A.E. (2017). Scattering Invisibility With Free-Space Field Enhancement of All-Dielectric Nanoparticles. Laser Photonics Rev..

[8] Zhou C., Li S., Fan M., Wang X., Xu Y., Xu W., Xiao S., Hu M., Liu J. (2020). Optical radiation manipulation of Si-*Ge*_2_*Sb*_2_*Te*_5_ hybridmetasurfaces. Opt. Express.

[9] Huang L., Xu L., Woolley M., Miroshnichenko A.E. (2020). Trends in Quantum Nanophotonics. Adv. Quantum Technol..

[10] Tian J., Li Q., Yang Y., Qiu M. (2016). Tailoring unidirectional angular radiation through multipolar interference in a single-element subwavelength all-dielectric stair-like nanoantenna. Nanoscale.

[11] Koshelev K., Kivshar Y. (2020). Dielectric resonant metaphotonics. ACS Photonics.

[12] Kruk S., Kivshar Y. (2017). Functional Meta-Optics and Nanophotonics Governed by Mie Resonances. ACS Photonics.

[13] Yang Q., Kruk S., Xu Y., Wang Q., Srivastava Y.K., Koshelev K., Kravchenko I., Singh R., Han J., Kivshar Y. (2020). Mie-resonant membrane huygens’ metasurfaces. Adv. Funct. Mater..

[14] Terekhov P.D., Baryshnikova K.V., Artemyev Y.A., Karabchevsky A., Shalin A.S., Evlyukhin A.B. (2017). Multipolar response of nonspherical silicon nanoparticles in the visible and near-infrared spectral ranges. Phys. Rev. B.

[15] Balezin M., Baryshnikova K.V., Kapitanova P., Evlyukhin A.B. (2018). Electromagnetic properties of the Great Pyramid: First multipole resonances and energy concentration. J. Appl. Phys..

[16] Yang Y., Miroshnichenko A.E., Kostinski S.V., Odit M., Kapitanova P., Qiu M., Kivshar Y.S. (2017). Multimode directionality in all-dielectric metasurfaces. Phys. Rev. B.

[17] Liu T., Xu R., Yu P., Wang Z., Takahara J. (2020). Multipole and multimode engineering in Mie resonance-based metastructures. Nanophotonics.

[18] Evlyukhin A.B., Tuz V.R., Volkov V.S., Chichkov B.N. (2020). Bianisotropy for light trapping in all-dielectric metasurfaces. Phys. Rev. B.

[19] Staude I., Pertsch T., Kivshar Y.S. (2019). All-Dielectric Resonant Meta-Optics Lightens up. ACS Photonics.

[20] Sayanskiy A., Kupriianov A.S., Xu S., Kapitanova P., Dmitriev V., Khardikov V.V., Tuz V.R. (2019). Controlling high-Q trapped modes in polarization-insensitive all-dielectric metasurfaces. Phys. Rev. B.

[21] Tuz V.R., Khardikov V.V., Kivshar Y.S. (2018). All-Dielectric Resonant Metasurfaces with a Strong Toroidal Response. ACS Photonics.

[22] Tian J., Luo H., Li Q., Pei X., Du K., Qiu M. (2018). Near-Infrared Super-Absorbing All-Dielectric Metasurface Based on Single-Layer Germanium Nanostructures. Laser Photonics Rev..

[23] Sayanskiy A., Danaeifar M., Kapitanova P., Miroshnichenko A.E. (2018). All-Dielectric Metalattice with Enhanced Toroidal Dipole Response. Adv. Opt. Mater..

[24] Gao Y., Fan Y., Wang Y., Yang W., Song Q., Xiao S. (2018). Nonlinear Holographic All-Dielectric Metasurfaces. Nano Lett..

[25] Jahani S., Jacob Z. (2016). All-dielectric metamaterials. Nat. Nanotechnol..

[26] Kuznetsov A.I., Miroshnichenko A.E., Brongersma M.L., Kivshar Y.S., Luk’yanchuk B. (2016). Optically resonant dielectric nanostructures. Science.

[27] Tuz V.R., Yu P., Dmitriev V., Kivshar Y.S. (2020). Magnetic Dipole Ordering in Resonant Dielectric Metasurfaces. Phys. Rev. Appl..

[28] Huang L., Yu Y., Cao L. (2013). General modal properties of optical resonances in subwavelength nonspherical dielectric structures. Nano Lett..

[29] Hasebe H., Sugimoto H., Hinamoto T., Fujii M. (2020). Coupled Toroidal Dipole Modes in Silicon Nanodisk Metasurface: Polarization Independent Narrow Band Absorption and Directional Emission. Adv. Opt. Mater..

[30] Jeong P.A., Goldflam M.D., Campione S., Briscoe J.L., Vabishchevich P.P., Nogan J., Sinclair M.B., Luk T.S., Brener I. (2020). High quality factor toroidal resonances in dielectric metasurfaces. ACS Photonics.

[31] Zhang J., MacDonald K.F., Zheludev N.I. (2013). Near-infrared trapped mode magnetic resonance in an all-dielectric metamaterial. Opt. Express.

[32] Terekhov P.D., Evlyukhin A.B., Redka D., Volkov V.S., Shalin A.S., Karabchevsky A. (2020). Magnetic Octupole Response of Dielectric Quadrumers. Laser Photonics Rev..

[33] Jain A., Moitra P., Koschny T., Valentine J., Soukoulis C.M. (2015). Electric and magnetic response in dielectric dark states for low loss subwavelength optical meta atoms. Adv. Opt. Mater..

[34] Yu P., Kupriianov A.S., Dmitriev V., Tuz V.R. (2019). All-dielectric metasurfaces with trapped modes: Group-theoretical description. J. Appl. Phys..

[35] Hu J., Lang T., Hong Z., Shen C., Shi G. (2018). Comparison of electromagnetically induced transparency performance in metallic and all-dielectric metamaterials. J. Light. Technol..

[36] Swiecicki S.D., Sipe J. (2017). Periodic Green functions for 2D magneto-electric quadrupolar arrays: Explicitly satisfying the optical theorem. J. Opt..

[37] Babicheva V.E., Evlyukhin A.B. (2019). Analytical model of resonant electromagnetic dipole-quadrupole coupling in nanoparticle arrays. Phys. Rev. B.

[38] Abujetas D.R., Olmos-Trigo J., Sáenz J.J., Sánchez-Gil J.A. (2020). Coupled electric and magnetic dipole formulation for planar arrays of particles: Resonances and bound states in the continuum for all-dielectric metasurfaces. Phys. Rev. B.

[39] Evlyukhin A.B., Poleva M.A., Prokhorov A.V., Baryshnikova K.V., Miroshnichenko A.E., Chichkov B.N. (2021). Polarization Switching Between Electric and Magnetic Quasi-Trapped Modes in Bianisotropic All-Dielectric Metasurfaces. Laser Photonics Rev..

[40] Chen W., Chen Y., Liu W. (2019). Multipolar Conversion Induced Subwavelength High-Q Kerker Supermodes with Unidirectional Radiations. Laser Photonics Rev..

[41] Liu W., Kivshar Y.S. (2018). Generalized Kerker effects in nanophotonics and meta-optics [Invited]. Opt. Express.

[42] Shamkhi H.K., Sayanskiy A., Valero A.C., Kupriianov A.S., Kapitanova P., Kivshar Y.S., Shalin A.S., Tuz V.R. (2019). Transparency and perfect absorption of all-dielectric resonant metasurfaces governed by the transverse Kerker effect. Phys. Rev. Mater..

[43] Miroshnichenko A.E., Evlyukhin A.B., Yu Y.F., Bakker R.M., Chipouline A., Kuznetsov A.I., Luk’yanchuk B., Chichkov B.N., Kivshar Y.S. (2015). Nonradiating anapole modes in dielectric nanoparticles. Nat. Commun..

[44] Liu S.D., Fan J.L., Wang W.J., Chen J.D., Chen Z.H. (2018). Resonance Coupling between Molecular Excitons and Nonradiating Anapole Modes in Silicon Nanodisk-J-Aggregate Heterostructures. ACS Photonics.

[45] Wu J., Zhang F., Li Q., Feng Q., Wu Y., Wu L. (2020). Strong Field Enhancement in Individual Φ Shaped Dielectric Nanostructures Based on Anapole Mode Resonances. Opt. Express.

[46] Wu P.C., Liao C.Y., Savinov V., Chung T.L., Chen W.T., Huang Y.W., Wu P.R., Chen Y.H., Liu A.Q., Zheludev N.I. (2018). Optical Anapole Metamaterial. ACS Nano.

[47] As’ ham K., Al-Ani I., Huang L., Miroshnichenko A.E., Hattori H.T. (2021). Boosting strong coupling in a hybrid WSe2 monolayer–anapole–plasmon system. ACS Photonics.

[48] Hsu C.W., Zhen B., Stone A.D., Joannopoulos J.D., Soljačić M. (2016). Bound states in the continuum. Nat. Rev. Mater..

[49] Lee S.G., Kim S.H., Kee C.S. (2020). Bound states in the continuum (BIC) accompanied by avoided crossings in leaky-mode photonic lattices. Nanophotonics.

[50] Xiang J., Xu Y., Chen J.D., Lan S. (2020). Tailoring the spatial localization of bound state in the continuum in plasmonic-dielectric hybrid system. Nanophotonics.

[51] Huang L., Xu L., Rahmani M., Neshev D., Miroshnichenko A.E. (2021). Pushing the limit of high-Q mode of a single dielectric nanocavity. Adv. Photonics.

[52] Abujetas D.R., de Sousa N., García-Martín A., Llorens J.M., Sánchez-Gil J.A. (2021). Active angular tuning and switching of Brewster quasi bound states in the continuum in magneto-optic metasurfaces. Nanophotonics.

[53] von Neumann J., Wigner E.P. (1929). Über merkwürdige diskrete Eigenwerte. Phys. Z..

[54] Kodigala A., Lepetit T., Gu Q., Bahari B., Fainman Y., Kante B. (2017). Lasing action from photonic bound states in continuum. Nature.

[55] Koshelev K., Favraud G., Bogdanov A., Kivshar Y., Fratalocchi A. (2019). Nonradiating photonics with resonant dielectric nanostructures. Nanophotonics.

[56] Yin X., Jin J., Soljačić M., Peng C., Zhen B. (2020). Observation of topologically enabled unidirectional guided resonances. Nature.

[57] Tian J., Li Q., Belov P.A., Sinha R.K., Qian W., Qiu M. (2020). High-Q all-dielectric metasurface: Super and suppressed optical absorption. ACS Photonics.

[58] Wang Y., Han Z., Du Y., Qin J. (2021). Ultrasensitive terahertz sensing with high-Q toroidal dipole resonance governed by bound states in the continuum in all-dielectric metasurface. Nanophotonics.

[59] Gorkunov M.V., Antonov A.A., Kivshar Y.S. (2020). Metasurfaces with maximum chirality empowered by bound states in the continuum. Phys. Rev. Lett..

[60] Abujetas D.R., Sánchez-Gil J.A. (2021). Near-Field Excitation of Bound States in the Continuum in All-Dielectric Metasurfaces through a Coupled Electric/Magnetic Dipole Model. Nanomaterials.

[61] Melik-Gaykazyan E., Koshelev K., Choi J.H., Kruk S.S., Bogdanov A., Park H.G., Kivshar Y. (2021). From Fano to quasi-BIC resonances in individual dielectric nanoantennas. Nano Lett..

[62] Koshelev K., Lepeshov S., Liu M., Bogdanov A., Kivshar Y. (2018). Asymmetric Metasurfaces with High-Q Resonances Governed by Bound States in the Continuum. Phys. Rev. Lett..

[63] Li S., Zhou C., Liu T., Xiao S. (2019). Symmetry-protected bound states in the continuum supported by all-dielectric metasurfaces. Phys. Rev. A.

[64] Wang X., Li S., Zhou C. (2020). Polarization-independent toroidal dipole resonances driven by symmetry-protected BIC in ultraviolet region. Opt. Express.

[65] Cao G., Dong S., Zhou L.M., Zhang Q., Deng Y., Wang C., Zhang H., Chen Y., Qiu C.W., Liu X. (2020). Fano resonance in artificial photonic molecules. Adv. Opt. Mater..

[66] Bogdanov A.A., Koshelev K.L., Kapitanova P.V., Rybin M.V., Gladyshev S.A., Sadrieva Z.F., Samusev K.B., Kivshar Y.S., Limonov M.F. (2019). Bound states in the continuum and Fano resonances in the strong mode coupling regime. Adv. Photonics.

[67] Tuz V.R., Khardikov V.V., Kupriianov A.S., Domina K.L., Xu S., Wang H., Sun H.B. (2018). High-quality trapped modes in all-dielectric metamaterials. Opt. Express.

[68] Overvig A.C., Malek S.C., Carter M.J., Shrestha S., Yu N. (2020). Selection rules for quasibound states in the continuum. Phys. Rev. B.

[69] Liu Z., Xu Y., Lin Y., Xiang J., Feng T., Cao Q., Li J., Lan S., Liu J. (2019). High-Q Quasibound States in the Continuum for Nonlinear Metasurfaces. Phys. Rev. Lett..

[70] Koshelev K., Tang Y., Li K., Choi D.Y., Li G., Kivshar Y. (2019). Nonlinear Metasurfaces Governed by Bound States in the Continuum. ACS Photonics.

[71] Koshelev K., Kruk S., Melik-Gaykazyan E., Choi J.H., Bogdanov A., Park H.G., Kivshar Y. (2020). Subwavelength dielectric resonators for nonlinear nanophotonics. Science.

[72] Han Z., Ding F., Cai Y., Levy U. (2020). Significantly enhanced second-harmonic generations with all-dielectric antenna array working in the quasi-bound states in the continuum and excited by linearly polarized plane waves. Nanophotonics.

[73] Xu L., Zangeneh Kamali K., Huang L., Rahmani M., Smirnov A., Camacho-Morales R., Ma Y., Zhang G., Woolley M., Neshev D. (2019). Dynamic Nonlinear Image Tuning through Magnetic Dipole Quasi-BIC Ultrathin Resonators. Adv. Sci..

[74] Volkovskaya I., Xu L., Huang L., Smirnov A.I., Miroshnichenko A.E., Smirnova D. (2020). Multipolar second-harmonic generation from high-Q quasi-BIC states in subwavelength resonators. Nanophotonics.

[75] Yesilkoy F., Arvelo E.R., Jahani Y., Liu M., Tittl A., Cevher V., Kivshar Y., Altug H. (2019). Ultrasensitive hyperspectral imaging and biodetection enabled by dielectric metasurfaces. Nat. Photonics.

[76] Zhou C., Qu X., Xiao S., Fan M. (2020). Imaging Through a Fano-Resonant Dielectric Metasurface Governed by Quasi–bound States in the Continuum. Phys. Rev. Appl..

[77] Chen Y., Zhao C., Zhang Y., Qiu C.W. (2020). Integrated Molar Chiral Sensing Based on High-Q Metasurface. Nano Lett..

[78] Ndao A., Hsu L., Cai W., Ha J., Park J., Contractor R., Lo Y., Kanté B. (2020). Differentiating and quantifying exosome secretion from a single cell using quasi-bound states in the continuum. Nanophotonics.

[79] Ha S.T., Fu Y.H., Emani N.K., Pan Z., Bakker R.M., Paniagua-Domínguez R., Kuznetsov A.I. (2018). Directional lasing in resonant semiconductor nanoantenna arrays. Nat. Nanotechnol..

[80] Cui C., Zhou C., Yuan S., Qiu X., Zhu L., Wang Y., Li Y., Song J., Huang Q., Wang Y. (2018). Multiple Fano Resonances in Symmetry-Breaking Silicon Metasurface for Manipulating Light Emission. ACS Photonics.

[81] Hwang M.S., Lee H.C., Kim K.H., Jeong K.Y., Kwon S.H., Koshelev K., Kivshar Y., Park H.G. (2021). Ultralow-threshold laser using super-bound states in the continuum. Nat. Commun..

[82] Sadrieva Z., Frizyuk K., Petrov M., Kivshar Y., Bogdanov A. (2019). Multipolar origin of bound states in the continuum. Phys. Rev. B.

[83] Zhou C., Liu G., Ban G., Li S., Huang Q., Xia J., Wang Y., Zhan M. (2018). Tunable Fano resonator using multilayer graphene in the near-infrared region. Appl. Phys. Lett..

[84] Zhou C., Li S., Wang Y., Zhan M. (2019). Multiple toroidal dipole Fano resonances of asymmetric dielectric nanohole arrays. Phys. Rev. B.

[85] Palik E.D. (1998). Handbook of Optical Constants of Solids.

[86] Cong L., Singh R. (2019). Symmetry-protected dual bound states in the continuum in metamaterials. Adv. Opt. Mater..

[87] Kyaw C., Yahiaoui R., Burrow J.A., Tran V., Keelen K., Sims W., Red E.C., Rockward W.S., Thomas M.A., Sarangan A. (2020). Polarization-selective modulation of supercavity resonances originating from bound states in the continuum. Commun. Phys..

[88] Mikheeva E., Koshelev K., Choi D.Y., Kruk S., Lumeau J., Abdeddaim R., Voznyuk I., Enoch S., Kivshar Y. (2019). Photosensitive chalcogenide metasurfaces supporting bound states in the continuum. Opt. Express.

